# Who should be undertaking population-based surveys in humanitarian emergencies?

**DOI:** 10.1186/1742-7622-4-12

**Published:** 2007-06-01

**Authors:** Paul B Spiegel

**Affiliations:** 1 United Nations High Commissioner for Refugees, Chief, Public Health and HIV Section, Division of Operational Support, PO Box 2500, CH 1211, Genève 2 Dépôt, Switzerland

## Abstract

**Background:**

Timely and accurate data are necessary to prioritise and effectively respond to humanitarian emergencies. 30-by-30 cluster surveys are commonly used in humanitarian emergencies because of their purported simplicity and reasonable validity and precision. Agencies have increasingly used 30-by-30 cluster surveys to undertake measurements beyond immunisation coverage and nutritional status. Methodological errors in cluster surveys have likely occurred for decades in humanitarian emergencies, often with unknown or unevaluated consequences.

**Discussion:**

Most surveys in humanitarian emergencies are done by non-governmental organisations (NGOs). Some undertake good quality surveys while others have an already overburdened staff with limited epidemiological skills. Manuals explaining cluster survey methodology are available and in use. However, it is debatable as to whether using standardised, 'cookbook' survey methodologies are appropriate. Coordination of surveys is often lacking. If a coordinating body is established, as recommended, it is questionable whether it should have sole authority to release surveys due to insufficient independence. Donors should provide sufficient funding for personnel, training, and survey implementation, and not solely for direct programme implementation.

**Summary:**

A dedicated corps of trained epidemiologists needs to be identified and made available to undertake surveys in humanitarian emergencies. NGOs in the field may need to form an alliance with certain specialised agencies or pool technically capable personnel. If NGOs continue to do surveys by themselves, a simple training manual with sample survey questionnaires, methodology, standardised files for data entry and analysis, and manual for interpretation should be developed and modified locally for each situation. At the beginning of an emergency, a central coordinating body should be established that has sufficient authority to set survey standards, coordinate when and where surveys should be undertaken and act as a survey repository. Technical expertise is expensive and donors must pay for it. As donors increasingly demand evidence-based programming, they have an obligation to ensure that sufficient funds are provided so organisations have adequate technical staff.

## Introduction

Timely and accurate data are necessary to prioritise interventions and effectively respond to humanitarian emergencies. Rapid initial assessments are essential first steps to help to establish whether a problem may exist. However, generally such 'quick and dirty' methods are not representative of the population. They should be quickly followed by the implementation of information systems comprised of facility-based surveillance systems supplemented by community-based reporting as well as population-based surveys.

Cluster surveys are commonly used in humanitarian emergencies because of their purported simplicity and reasonable validity and precision. These surveys are primarily undertaken to estimate nutrition and mortality outcomes among affected populations. They require only approximate estimates of the relative sizes of the population units sampled; no lists of individuals or households are necessary as with simple random or systematic sampling [1]. Cluster sampling methods were first used to assess immunisation coverage in developing countries [2], but also have been validated for estimating both immunisation coverage [2,3] and nutritional status [4,5].

The recommended standardised methodology for nutrition surveys consists of 2-stage sampling [6-8]. The first stage requires the grouping of the population into 30 smaller geographical units, or clusters, that are proportional to population size. The second stage requires the selection of households and then 30 children in each cluster from whom anthropometric measurements are taken; thus, the name 30-by-30 nutrition cluster survey. Agencies have increasingly used the 30-by-30 cluster survey methodology in humanitarian settings to undertake the measurements of outcomes beyond immunisation coverage and nutritional status, such as behaviour [9], morbidity [10,11] and mortality outcomes [12-18]; often these other measurements are included in immunisation and/or nutrition surveys. Such expanded use of the cluster survey methodology raises concern about the validity and precision of these various estimates.

Manuals that explain the 30-by-30 nutrition cluster survey methodology, step-by-step, are available and widely used in the field [6-8]. However, despite the reported simplicity of conducting cluster surveys, there is increasing evidence that methodological errors in cluster surveys conducted in humanitarian emergencies are likely to have occurred for decades, thereby resulting in inaccurate estimates of the prevalence of the outcome being surveyed. The consequences of using inaccurate or poorly obtained survey data to prioritise programme implementation and funding are unknown. During the Somali famine in 1991/92, Boss et al. evaluated 16 nutrition surveys and found a lack of standardisation in methodology [19]. Garfield described 27 nutritional surveys in Iraq during the 1990s and found them to be of uneven quality [20]. My colleagues and I evaluated 125 surveys by 14 non-governmental organisations (NGOs) during the famine in Ethiopia during 1999/2000 and found major methodological errors [21]. Recently, we evaluated 31 HIV behavioural surveillance surveys in emergency and post-emergency situations (1998–2005) using cluster methodology in 14 countries and again found significant methodological errors; the surveys undertaken by NGOs were significantly less reproducible than those undertaken by non-NGOs [9]. The major methodological errors in all of these reviews included insufficient sample size or number of clusters, failure to sample proportional to population size in stage one, failure to weigh the sample during analysis when only one eligible person per household was chosen, and failure to consider the design effect when calculating precision.

For example, in Ethiopia during 1999/2000, there was insufficient central coordination of nutrition surveys. It was unclear who was undertaking nutrition surveys or where they were being done. Furthermore, there was no oversight of the quality or interpretation of these surveys. Thus, surveys by different NGOs sometimes occurred in overlapping geographical areas while none were being undertaken in other areas. Rapid, non-probabilistic (so called 'convenience' surveys) occurred in some areas where they were often not followed by probabilistic nutrition surveys measuring weight-for-height. While some organisations undertook excellent nutrition surveys with insightful analysis followed by repeat surveys to evaluate the effectiveness of their interventions, others undertook cluster surveys but did not sample proportional to population size and, therefore, likely produced biased and non-representative results. Donors then used the results of these surveys to provide hundreds of millions of dollars of funding. Although it is not possible to know the consequences of such actions, donor funds are limited and it is likely that the insufficient or inaccurate data reduced the effectiveness, efficiency and equity of the response.

There are numerous criticisms regarding cluster survey methodology and many suggestions on how to improve it [1,7,22-25]. Furthermore, some researchers are comparing the results from cluster methodology to other methods of measurement in order to assess its validity and precision [26,27]. This thematic issue on surveys in *Emerging Themes of Epidemiology *will be presenting both first- and second-stage sampling issues in more detail. I wish to discuss something more fundamental – *Who should be undertaking population-based surveys in humanitarian emergencies?*

## Discussion

Most of the surveys undertaken in humanitarian emergencies are by NGOs. Many of the errors occurring during the survey design and data analysis phases can be prevented by using properly trained technical personnel in the field at the time of the surveys. While some NGOs have trained epidemiologists who undertake high quality surveys, others have a paucity of staff with insufficient epidemiological skills to undertake such surveys. Furthermore, persons in the field are already overburdened with existing responsibilities and are unable to add an additional complicated, technical and time-consuming task such as a survey. Even with increased training of NGO staff on survey methodology and analysis, the high turn-over of staff in these circumstances make it a lengthy and expensive proposition that must be constantly repeated.

A dedicated corps of trained epidemiologists needs to be identified and made available to undertake these surveys. This will take some time to develop, since most NGOs do not have sufficient funds to train and maintain such a cadre. In the short term, NGOs in the field may need to form an alliance with certain other specialised NGOs or other agencies whose main focus is surveys. This may be the long term solution for those NGOs that do not wish to or are not able to identify and maintain a team of trained epidemiologists. Another option would be to have some agencies send technically capable personnel to the emergency that can be utilised by other NGOs to undertake such surveys. However, both solutions require a level of coordination and cooperation that is not frequently seen in the field. Furthermore, funds specifically designated for this purpose must be made available by donors.

There is a debate as to whether it is appropriate to use standardised survey methodologies at all. This so-called 'cookbook' approach was developed for non-epidemiologists to undertake population-based surveys to measure immunisation coverage and nutritional status. However, a 30-by-30 cluster survey may not be needed in many situations and may be insufficient in other circumstances. An epidemiologist should be able to choose an appropriate sampling methodology (e.g. sometimes cluster survey methodology is used when simple or systematic sampling could be employed; the latter has a smaller sample size and thus can save time and money for the same precision), calculate appropriate sample sizes, use a design effect other than 2.0, which is often used for nutrition and other cluster surveys, and choose an adequate number of clusters and households or persons per cluster that may differ from the 30-by-30 approach. Reducing the overall sample size and adjusting the number of clusters and households or persons per cluster in order to have sufficient precision may improve the accuracy of the data collected by allowing the hiring of fewer, but better qualified, surveyors with improved supervision. This strategy will also save money and, particularly, time, which can be critical in humanitarian emergencies where surveys often occur in unstable and dangerous situations [24]. Conversely, increased sample sizes are required to measure rare outcomes compared with those required for more common outcomes.

If a cadre of epidemiologists or a specialised agency, as suggested above, is sent to the field and coordinates the surveys, a cookbook approach to undertaking surveys is not necessary. However, if NGOs continue to do surveys by themselves, more can be done to ensure that the previously documented methodological errors are reduced. The 30-by-30 cluster survey methodology is sufficiently well-documented in many commonly used field manuals and likely cannot be further simplified without affecting its validity and precision. However, a sample survey questionnaire translated into local languages with an events calendar should be developed as a template for all agencies to use during each emergency. A training manual that includes sampling methodology that has been modified according to the context of the crisis could quickly be developed. Furthermore, standardised files for data entry as well as programmes for analysis and a manual for interpretation could also be developed and shared. Such a standardised approach, if sufficient coordination is in place, would allow for improved quality as well as comparability of surveys.

Besides conducting technically sound surveys, coordination and cooperation of surveys by governments, United Nations (UN) agencies and NGOs in humanitarian emergencies are essential. At the beginning of an emergency, a central coordinating body should be established that has sufficient authority to, at minimum, set survey standards, coordinate when and where surveys should be undertaken and by whom, and to act as a survey repository. It is debatable whether this coordinating body should have the sole authority to review and disseminate surveys. Depending upon the composition and competence of the coordinating body, such authority has obvious benefits. However, humanitarian emergencies are inherently political. Coordinating bodies may be controlled by governments who may not wish to release surveys that place their administrations in a bad light. Furthermore, UN agencies and other organisations are often reliant upon governments and may not wish to release information that will affect their standing in the country if the government does not wish the survey results to be released. This was likely the case with respect to recent mortality surveys in Darfur and in Northern Uganda. Thus, the authority and composition of the coordinating body must be carefully decided upon with political considerations, unfortunately, being an essential element. Ultimately, all surveys, including the original data with all identifying factors removed, should be available on the internet to ensure accessibility and transparency. A successful example of a strong and transparent coordinating body is the Food Security Analysis Unit in Somalia. This unit seeks to provide evidence-based analysis of Somali food, nutrition and livelihood security to enable both short-term emergency responses and long-term strategic planning to promote food and livelihood security for Somali people [28].

Identifying who should set standards, identify a corps of epidemiologists, develop a training manual(s) and provide coordination leadership as well as decide where a survey repository should be housed is a controversial issue. Each humanitarian emergency is different and has unique aspects that would influence these decisions. Thus, except possibly for standard setting, I do not believe a global body should be established. Rather, at the beginning of each emergency, a technical and political assessment should be immediately undertaken to decide which organisation(s) would be best placed to provide such coordination leadership as well as the composition and authority of such a coordinating body. Numerous courses and degrees already exist to train epidemiologists on how to undertake surveys in humanitarian emergencies. The development of one recognised and accredited training course is unrealistic and unnecessary. The need for technical expertise does not impact only governments, UN agencies and NGOs. Donors need to establish a mechanism to ensure that they have the means to evaluate the quality and the interpretation of the surveys that they receive from NGOs and other organisations. Such a mechanism was clearly not in place during the 1999/2000 famine in Ethiopia where poorly conducted surveys influenced policy and resource allocation [21]. Donors do not necessarily need to have staff in-country during the crisis, but they should at least have access to and use technical expertise to inform their decision making. For such an arrangement to be successful, survey reports will need to be composed in a more comprehensive, systematic and detailed manner than is often done. A competent centralised coordinating body for each emergency, as discussed above, would solve this problem. Otherwise, if donors continue to receive survey reports from individual agencies, these reports should be reviewed by competent and experienced epidemiologists. Given the widespread access to internet in most settings these days, this is eminently feasible.

Technical expertise is expensive. Donors must clearly recognise this fact and pay for it. Many donors prefer to fund direct programme implementation rather than salaries and trainings which they consider indirect or administrative costs that divert funds from the beneficiaries. However, with the increasing demand from donors for evidence-based programming, donors have an obligation to ensure that sufficient funds are provided to organisations so they have sufficient technical staff and training to provide the evidence they require. Accurate survey data and appropriate technical guidance (e.g. staff, guidelines and training) is essential to ensure effective humanitarian response. Ultimately, funding technical expertise may save money as good quality surveys should allow for interventions to be designed, implemented and targeted much more efficiently and effectively.

In the response to humanitarian emergencies to date, there has often been poor quality surveys, insufficient coordination, political interference and inadequate funding for the provision of reliable and timely survey information. The effects of these insufficiencies are unknown. However, it is likely that policies, programmes and hundreds of millions of dollars in resource allocation have been decided upon, at least in part, using bad information. The recommendation that NGOs develop sufficient expertise or have sufficient funding to pay for such expertise to ensure adequate surveys are undertaken in humanitarian emergencies should not necessarily be seen as a suggestion for NGOs to move into the research field. Rather, surveys in humanitarian emergencies should be seen as an essential and basic component of every programme to ensure adequate data are available to target interventions and to measure their effectiveness. A centralised coordinating body developed at the onset of each humanitarian emergency together with sufficient funding for a cadre of competent epidemiologists will not solve all of these problems. However, it would be a good start.

## Competing interests

The author(s) declare that they have no competing interests.

## Endnote

This manuscript is a personal viewpoint that does not necessarily represent the views of the United Nations High Commissioner for Refugees.
